# Headgear use in girls’ lacrosse—stakeholders not ready for change

**DOI:** 10.3389/fspor.2024.1363007

**Published:** 2024-06-04

**Authors:** Robyn Recker, Alison Myers, Nikhil Desai, Jaclyn B. Caccese, Laura Boucher, James Onate, Jingzhen Yang

**Affiliations:** ^1^ Center for Injury Research and Policy, The Abigail Wexner Research Institute at Nationwide Children’s Hospital, Columbus, OH, United States; ^2^ School of Health & Rehabilitation Sciences, The Ohio State University College of Medicine, Columbus, OH, United States; ^3^ Sports Medicine Research Institute, The Ohio State University College of Medicine, Columbus, OH, United States; ^4^ Division of Athletic Training, The Ohio State University, Columbus, OH, United States; ^5^ Department of Pediatrics, The Ohio State University College of Medicine, Columbus, OH, United States

**Keywords:** clinician, coach, concussion, helmet, injury prevention, officials, policy development

## Abstract

**Purpose:**

Mandating headgear for field players in girls’ lacrosse to reduce head injuries, including concussion, has been heavily debated. However, research regarding the need and effectiveness of mandated headgear use in girls’ lacrosse is still developing. Therefore, this qualitative study aimed to identify the need for and barriers to the development of mandated headgear use policies in girls’ lacrosse in Ohio.

**Methods:**

We conducted six virtual focus groups, three with concussion experts (clinicians and researchers) and three with girls’ lacrosse stakeholders (high school players, parents, coaches, and officials). A focus group guide was developed to explore study participants’ perceptions and opinions on concussion in girls’ lacrosse, headgear use among players, and policies and policy development related to headgear use or a headgear mandate. We developed the codebook using an inductive and iterative approach based on focus group transcripts and used ATLAS.TI to code and analyze the transcript data.

**Results:**

Concussion experts and stakeholders understood the potential consequences of concussion but did not perceive concussion as a pervasive problem in girls’ lacrosse. The prevention of head and facial injuries was regarded as a potential benefit of headgear use. However, stakeholders expressed that the myriad of arguments discussed opposing mandated headgear use including increased aggressive play and/or targeting, concerns over changes in the game, and cost strongly outweighed the benefits. Finally, both concussion experts and stakeholders identified multiple organizations, including USA Lacrosse, who could act as facilitators and/or barriers to developing, enacting, and implementing headgear policies.

**Conclusions:**

Concussion experts and stakeholders identified possible reasons for headgear use related to injury prevention but also identified several important barriers to the development of a headgear mandate for girls’ lacrosse in Ohio.

## 1 Introduction

Lacrosse is one of today's fastest-growing youth sports in the US, with a 226% increase in national participation across all ages from 2001 to 2017 (1). Further, high school girls’ lacrosse has also experienced rapid growth with a 54% increase from 2009 to 2019 (2, 3). Although the basic concepts and scoring are similar between boys’ and girls’ lacrosse, the rules vary substantially. While checking in the head or neck area is prohibited in both boys’ and girls’ lacrosse, boys’ lacrosse allows both body and stick checking but requires all players to wear a hard-shell helmet with a full facemask, shoulder pads, a National Operating Committee on Standards for Athletic Equipment (NOCSAE) chest protector, elbow pads, and gloves to protect from player-to-player and stick and ball contact (4). Conversely, girls’ lacrosse relies more heavily on rules, such as prohibiting body checking and penalties for entering the head sphere, rather than equipment for player protection. Aside from the goalkeeper, players are only mandated to wear NOCSAE protective eyewear, but field players are permitted to wear flexible-shelled headgear with a partial face mask for additional protection (4–6).

Evidence suggests concussion rates are as high as 3.91 per 10,000 athlete-exposures for high school girls’ lacrosse players from 2008 to 09 through 2018-19 academic year (5). While player-to-player contact is the primary mechanism of concussion for boys, accounting for 66.4% of all concussions, stick or ball contact is the primary mechanism of concussion for girls, accounting for 72.7% of all concussions (5, 7, 8). Concussion rates from stick or ball contact are 2.6 times higher among high school girls’ lacrosse players compared to high school boys’ lacrosse players (5, 9–11). These findings on the rates and mechanisms of injury have led to a debate over headgear usage and/or mandate for field players in girls’ lacrosse (7). Supporters of headgear use in girls’ lacrosse believe it could reduce the risk of concussion resulting from stick and ball contact. However, questions remain unanswered as to whether the addition of headgear to girls’ lacrosse would increase the risk of concussion and/or other injuries from body contact.

While there has been resistance to the adoption of headgear in the girls’ lacrosse community, some evidence of the benefits of headgear has begun to emerge. Though headgear does not prevent all concussions, some evidence suggests that headgear might provide some level of protection from concussion amongst girls’ lacrosse players (5, 7). In 2018, Florida became the first state to mandate headgear use in high school girls’ lacrosse. Following the enactment of the mandate, concussion rates were 59% lower in Florida (2.7 per 10,000 athlete-exposures) than in states without headgear requirements (4.4 per 10,000 athlete-exposures) despite similar rates of musculoskeletal injury (12, 13).

Headgear use mandates have not yet been implemented in other states, including Ohio. Given the high incidence of concussion in girls’ lacrosse, along with the potential for long-term impairment from brain injury (14, 15), it is critical to support brain injury prevention efforts in girls’ lacrosse (5, 8, 9, 16). One next step to support these efforts could be policy development related to headgear in girls’ lacrosse. However, much is still unknown about the headgear needs of the girls’ lacrosse community. Therefore, the purpose of the present study was to identify the need for and barriers to the development of mandated headgear use policies in girls’ lacrosse in Ohio.

## 2 Methods

### 2.1 Participants and study design

We conducted focus groups with participants from two areas: (1) concussion experts, including concussion researchers who had published 5 or more peer-reviewed manuscripts on pediatric concussions and clinicians who had provided concussion care to 10 or more pediatric concussion patients, and (2) members of the girls’ lacrosse community in Ohio, including current/former girls’ lacrosse players, aged 11 or older and played at least one season in the past 12 months, parents of players, coaches, and officials of girls’ lacrosse in Ohio in the same age range during the past 12 months. Additional inclusion criteria were the ability to comprehend and speak English.

### 2.2 Recruitment and data collection

We recruited focus group participants via email, research participant database (i.e., ResearchMatch), word of mouth, and snowball sampling. Specifically, recruitment was initiated with emails to groups of concussion experts (e.g., sports-medicine physicians) and lacrosse-focused organizations in the community including girls’ youth and high school lacrosse leagues and officials’ organizations. Interested individuals were contacted to be scheduled for potential focus groups.

Prior to participation in the focus group, all participants reviewed and signed a consent/assent form and completed a demographics survey. Focus groups were conducted virtually on a secure platform and were audio recorded for transcription purposes. Each focus group lasted 45–60 min and was facilitated by two researchers, one with experience in pediatric sports concussion research and one with knowledge of lacrosse. This study was approved by the Institutional Review Boards of the participating institutions. This report follows the Standards for Reporting Qualitative Research (SRQR) (17).

We conducted six focus groups, three with concussion experts (clinicians and researchers) and three with girls’ lacrosse stakeholders (players, parents, coaches, and officials), with 5–9 individuals per group. Players were in a separate focus group from their parents and coaches to increase comfort of participants and reduce possible conformity bias.

### 2.3 Development of focus group guide

A focus group guide was developed by the research team to explore study participants’ perceptions and opinions on (1) Experience with girls’ lacrosse and concussion; (2) General thoughts about girls’ lacrosse headgear; (3) Whether Ohio should require headgear for girls’ lacrosse; (4) Facilitators and barriers to policies regarding headgear in girls’ lacrosse; and (5) Thoughts on policy development related to headgear in girls’ lacrosse. A written draft of the focus group guide was shared with concussion experts and members of the girls’ lacrosse community in Ohio outside of the research team and the content of questions was revised accordingly prior to data collection. Separate, but very similar guides were used for the focus groups discussions with the concussion experts and the stakeholders to gain insights into each group's perspectives, experiences and opinions regarding the need for and contextual barriers to the development a headgear mandate for girls’ lacrosse in Ohio.

A brief introduction script was developed to remind participants of the study purpose and confirm their consent/assent to participation and audio recording. For the concussion expert focus groups, an additional introduction on basic concepts about girls’ lacrosse and the allowable headgear (e.g., flexible design to absorb impact from stick or ball) was provided, accompanied with images of the required helmet for boys and the optional headgear for girls along with statistics regarding the most common concussion mechanisms among girls (e.g., ball/stick contact). The purpose of this was to provide background information as not all of the concussion experts had direct experience with girls’ lacrosse.

### 2.4 Data analysis

Audio recordings from each focus group were transcribed verbatim using a third-party transcription service. Following transcription, before coding, the first author used an inductive and iterative approach to develop the initial codebook based on two transcripts, one from a concussion expert focus group and one from a stakeholder focus group. This initial codebook was used by two other researchers to code the same two transcripts, then reviewed and discussed and collectively agreed to the coding definition, inclusion criteria, and exclusion criteria by the research team before being finalized. The transcripts and finalized codebook were then imported into a qualitative analysis software, ATLAS.TI and independently coded by three research team members. All the members of the research team met to compare themes for consistency, with any discrepancies resolved through consensus during team meetings, after which a thematic analytical approach was used to organize the resulting codes into themes (18, 19).

## 3 Results

### 3.1 Participants

A total of 40 participants were included in the focus groups, including 19 concussion experts and 21 stakeholders (9 players, 12 parents, coaches, and/or officials). The concussion experts were primarily male (*n* = 12, 63.2%) with an average age of 42.8 years (Table 1). Players were all female with an average age of 17.4 years old and parents, coaches, and officials were primarily female (*n* = 9, 75.0%) with an average age of 48.7 years old (Table 1).

**Table 1:** Participant demographics.

Variable	Concussion Experts (*N* = 19)	Players (*N* = 9)	Parents, coaches and officials (*N* = 12)
	Mean (SD) or *N* (%)	Mean (SD) or *N* (%)	Mean (SD) or *N* (%)
Sex			
Male	12 (63.2%)	0 (0%)	3 (25.0%)
Female	7 (36.8%)	9 (100%)	9 (75.0%)
Age	42.8 (10.7)	17.4 (1.7)	48.7 (9.4)
Education			
Some high school	0 (0%)	5 (55.6%)	0 (0%)
High school graduate	0 (0%)	1 (11.1%)	0 (0%)
Some college	0 (0%)	3 (33.3%)	1 (8.3%)
Four-year college Graduate	1 (5.3%)	0 (0%)	7 (58.3%)
Some graduate school or Higher	17 (89.5%)	0 (0%)	4 (33.3%)
Not Reported	1 (5.3%)	0 (0%)	0 (0%)
Role^a^			
Research	3 (15.8%)		
Research & clinician	3 (15.8%)		
Clinician—physician	4 (21.0%)		
Clinician—physical therapist	4 (21.0%)		
Clinician—other	1 (5.3%)		
Player		9 (100%)	
Parent			7 (58.3%)
Coach			5 (41.7%)
Official			3 (25.0%)
Other^b^			2 (16.7%)

^a^ Individual could identify with more than one role.

^b^ Other roles included club owner/director and community youth coordinator.

### 3.2 Themes and codes

Themes and codes fell under one of three topics (1) Perceptions of concussion: participants’ comprehension or understanding of concussion, (2) Attitudes towards headgear use or mandate: participants’ feeling or way of thinking about headgear use or a mandate or (3) Policy: participants’ thoughts on the development, enactment, and implementation of headgear mandate policies for girls’ lacrosse. One theme, perception of concussion, emerged under the topic of perceptions. Under attitudes four themes emerged including player safety and injury, aggressiveness, culture, and physical characteristics. Finally, for policy, five themes emerged related to player characteristics, enacting or implementing headgear mandates, supports needed, or strategical approaches for girls’ lacrosse players in Ohio. A list of themes and codes along with definitions is in Table 2.

**Table 2:** Topics, themes, codes, and definitions.

Topic	Themes	Codes	Definitions
Perceptions	Perception of concussion	Concussion	Discussion of concussion including risk, incidence, history of, consequences of, and experience
		Concussion in girls’ lacrosse	Discussion of concussion risk, incidence, and mechanisms in girls’ lacrosse
Attitudes	Player safety and injury	Safety concerns during play	Discussion of safety concerns in girls’ lacrosse, includes how headgear could increase player safety
		Head/facial injury prevention	Discussion of the role of headgear in preventing head and/or facial injuries
	Aggressiveness	Increased aggression	Discussion about increased aggression due to headgear use in general or specifically by players wearing headgear
		Players targeted	Discussion about players who wear headgear being targeted during play
	Culture of the sport	Change in the game of girls’ lacrosse	Discussion about headgear changing the way the game of girls’ lacrosse is played
		Self-expression	Discussion about headgear inhibiting self-expression
		Cost	Discussion about the high cost of equipment, including headgear, or increased cost to participate if headgear was mandatory
	Physical characteristics	Comfort	Discussion about the comfort of headgear
		Vision concerns	Discussion about headgear leading to decreased vision of the field
Policy	Player characteristics	Age	Discussion about headgear use policies based on age of players
		Skill level	Discussion about headgear use policies based on skill level of players
	Enacting or implementing a headgear mandate	Organization as a facilitator	Discussion of an organization and/or its characteristics acting as a facilitator for enacting or implementing headgear use policies or mandate
		Stakeholder as a facilitator	Discussion of an individual or group of individuals acting as a facilitator for enacting or implementing headgear use policies or mandate
		Organizations as a barrier	Discussion of an organization and/or its characteristics acting as a barrier to enacting or implementing headgear use policies or mandate
		Stakeholder as a barrier	Discussion of an individual or group of individuals acting as a barrier to enacting or implementing headgear use policies or mandate
	Supports needed for promoting use or mandate	Stakeholder support	Discussion of headgear policies requiring support from an individual, group of individuals, or organization to enact or implement
		Additional evidence	Discussion about the desire for additional evidence to support headgear use policies or mandate
	Strategical approaches	Focus on other injuries	Discussion about focusing the support for headgear use policies or mandate on injuries other than concussion, such as facial or head lacerations
		Optional use	Discussion of the current optional nature of headgear use among girls’ lacrosse players

### 3.3 Perceptions of concussion

Stakeholders, including players, expressed their understanding that concussions are a prevalent issue in sports that can have serious consequences. However, they did not view concussion as a large problem in girls’ lacrosse.

*“I think that concussions are an issue in any sport. No matter what sport you play, if there's contact, there's like a chance of getting a concussion.” – Player*

*“I've seen a ton of lacrosse and the girls have played a ton of lacrosse and, again, not saying [concussion's] not out there, I haven't seen it at all.” – Parent*

This view on concussion consequences in girls’ lacrosse may be influenced by the relatively low prevalence of lacrosse-related concussion experienced by participants as only one player reported sustaining a concussion while playing lacrosse, while no parents reported their child had sustained a concussion playing girls’ lacrosse. Of the coaches, three reported having an athlete on their team who had sustained a concussion playing girls’ lacrosse. Two officials reported having an athlete sustain a concussion while officiating a girls’ lacrosse game.

Concussion experts voiced similar thoughts as stakeholders and some expressed surprise that ball or stick contact was the most common mechanism of concussion among girls’ lacrosse players, with a clinician stating, “I very rarely ever see [a concussion] from the gameplay as in a ball or a stick hitting a girl and getting hit.”

### 3.4 Attitudes towards headgear use or mandate

#### 3.4.1 Arguments in favor of headgear use or mandate

Increased player safety was the main reason for stakeholders supporting the use of headgear or a headgear mandate.

*“I feel like safety-wise, the goggles don't really do much. The helmet would do much more.” – Player*

Similarly, concussion experts felt the use of headgear, or a headgear mandate would increase the safety of players and prevent head and/or facial injuries.

*“There's so many different head injuries that can happen beyond concussion that a helmet would potentially protect.” – Clinician*

#### 3.4.2 Arguments opposing headgear use or mandate

Stakeholders and concussion experts identified several reasons for opposing headgear use including increased aggressive play, targeting of players who wear headgear, concerns it would lead to changes in the way that girls’ lacrosse is played, restricted vision, and cost. In particular, coaches and officials were concerned about increased aggression and how this increased aggression would negatively change the game.

*“There's an increased physicality whether it is the player that is wearing the headgear, or a defender or opponent going after the individual with headgear a little bit more often.” – Coach*

*“The aggressiveness at the third, fourth-grade level even, if you're looking at that young level, how much more aggressive they get when they have that headgear on shows me right away that that is not how we want the sport to grow and to go.” – Official*

Additionally, players spoke about targeting players on opposing teams who wore headgear, with a player stating “We played a team, and one girl had a helmet, we voted her as the weak link. We all went after her and were a little more aggressive with her.” Some players also stated while they were not opposed to the increased aggression occurring across the sport of girls’ lacrosse, they enjoyed that their game was still different from boys’ lacrosse and did not want their sport to “turn into the boys’ game” or require pads.

While some of the concussion experts did not have the first-hand experience to comment directly on the aggressiveness or changes in play in girls’ lacrosse, some stated they were aware of these arguments or had heard these from members of the lacrosse community.

*“I do know the background on this is that I have heard stories where having helmets affects kids, girls’ willingness to be more aggressive.” – Researcher*

*“So that's where I've heard coaches say they don't want the helmets because then it's going to make it an even rougher game, because the girls are going to start swinging the sticks harder at the other player stick to try to knock the ball out and cause more injury just having a helmet.” – Clinician*

Stakeholders also expressed concerns over restricted vision and movement when wearing the headgear, as a coach mentioned, “For the most part the girls don't want to wear them [headgear] because of the ability to see and move their heads quickly. I've heard their field of vision is sometimes affected by them.” Moreover, stakeholders and concussion experts alike voiced apprehensions related to the cost of this added equipment and how that may limit opportunities for athletes, especially for those who lack financial resources, to try the sport.

*“I wouldn't consider lacrosse an inexpensive sport and if you mandate an extra piece of equipment, that is an extra cost that will have to be updated throughout the years” – Coach*

*“Some of these other school districts that don't have as much money, but they have an interest in having that lacrosse program, they're going to be like, “Well, it costs too much now for us to cover that so we're not going to offer that sport.” So now we're potentially taking a sport away from some young female that wants to try it.” – Clinician*

Finally, many players also felt the headgear would be uncomfortable and may prevent self-expression while playing, for example by covering up intricate hairstyles which they reported as common in the culture of girls’ lacrosse.

*“I feel it would be all hot and sweaty in that and make you feel really sweat on your face dripping down into your eyes.” – Player*

*“My team is very into the different hairstyles, which makes getting ready for games fun and brings the team together. We'll be in the locker room, and there's people doing each other's hair and helping each other out. With the helmet, it just covers up the hairstyles that they do.” – Player*

### 3.5 Headgear mandate policies for girls’ lacrosse

#### 3.5.1 Proposed mandate by characteristics of players

Stakeholders and concussion experts proposed that a mandate should be based on characteristics of the players, such as age and/or skill level. Most proposed the mandate be for younger and/or less skilled players, mostly due to concerns regarding lack of ball and/or stick control.

*“I also agree with the idea that younger kids have less control of their bodies, react less quickly, and they just don't make good decisions… So, I agree with more of [headgear use] as an obligation for the younger set” – Researcher*

*“I think they'd be more apt to be willing to put them on if they're not as experienced, if they're not as good of players” – Parent*

However, some players argued that headgear should only be required for older players, citing that these players may hit and/or swing harder than younger players, “In middle school, I feel like people don't hit hard enough and swing hard enough that it'd [headgear] be needed.”

Finally, some concussion experts mentioned concerns over headgear slowing player development if implemented among younger players, stating “If the youngest girls were just starting to play lacrosse are wearing a helmet, they're not quite going to develop as good of finesse and skills at that age as they would have if they didn't have the helmet on.” Notably, though there are progressive changes in the rules with age, gradually increasing the amount and changing placement of checking and body contact allowed, it is likely that most concussion experts and some stakeholders are not aware of these rule progressions.

#### 3.5.2 Facilitators to enacting or implementing mandate

Stakeholders discussed both organizations and the stakeholders themselves, which could serve as facilitators to enacting and implementing a headgear mandate for girls’ lacrosse players in Ohio. The organization's crucial role in facilitating a headgear mandate was described by a coach as,

*“I think that if [USA Lacrosse] were to be involved and to mandate, I think that a lot of their involvement could potentially be in things similar as this focus group, which would discuss more of the details of what a helmet could and could not benefit” – Coach*

Similarly, concussion experts expressed that both organizations, especially sports/medical organizations in the lacrosse community, could facilitate the enactment and implementation of a headgear mandate for girls’ lacrosse players in Ohio. For example, a clinician mentioned that the American Medical Society for Sports Medicine could facilitate a mandate by putting out a guideline recommending headgear for girls’ lacrosse athletes.

*“One other way to change policy too, … was the American Medical Society for Sports Medicine. They put out guidelines for concussion in general or for different sports. So, if they were to identify this as a problem, put out a guideline that, “As a Society of Sports Medicine Doctors, we recommend headgear use in girls’ Lacrosse.” I think that goes a long way.” – Clinician*

Importantly, while a recommended guideline from an organization of health professionals is not synonymous with mandates that are enforceable by a sport's governing body, these types of guidelines provide supporting evidence that can be presented to the governing bodies that have the ability to create and enforce mandates.

#### 3.5.3 Barriers to enacting or implementing mandate

Potential policy barriers mentioned across all the focus groups included many of the same organizations and stakeholders brought up as potential facilitators. In line with this thought, due to their own opposition of headgear, the stakeholders viewed themselves along with organizations they were involved in, such as the official's association throughout Ohio, as a major barrier to enacting and implementing a headgear mandate.

*“I would say [a barrier for] wearing headgear would be the officials’ association throughout the state.” – Official*

Concussion experts recognized that stakeholders’ motivations and actions would serve as a large barrier to a headgear mandate, with a clinician stating, “I think the biggest barrier is going to be those that it's going to affect most, … the players and the parents of those players.”

#### 3.5.4 Supports needed for promoting use or mandate

All focus group participants overwhelmingly agreed that the largest support needed for promoting headgear use or a headgear mandate was the demand for additional evidence regarding headgear's role in preventing and reducing head injuries in girls’ lacrosse to support a mandate.

*“I wonder about what Florida has put out there, defending this decision, or any longitudinal data that they have gathered over the years. … I'd like to see if there's data out there that they've published that talks about concussions or injuries related to headgear.” – Coach*

*“We do need science to inform this. We do need data. We have some in Florida. Is that enough to make a change?” – Clinician*

Additionally, concussion experts recognized that lacrosse organizations, athletes, and coaches would also play a more significant role in a headgear mandate, as stated by a researcher, “I think a national organization like [USA Lacrosse] that’s specific to the sport, I think probably carries a lot of weight with the athlete/coaches’ side of things.”

#### 3.5.5 Strategical approaches

The final theme that emerged was related to strategies for approaching a headgear mandate. Concussion experts, particularly clinicians, argued that the push for a mandate would be more successful if the messaging focused on other head injuries besides concussions, such as facial or head lacerations.

*“If we're considering a helmet for a sport, it's not just for a concussion that you would want to consider, it's the other injuries which are probably more common such as maybe contusions, lacerations to the head.” – Clinician*

*“From the perspective of reducing head and facial, oral-facial injuries, I think that's where a mandate, and if it's messaged the right way, with the things that are more often happening because there's not as many concussions, it's probably more of those things. I think it would be probably better received” – Clinician*

Concussion experts, specifically the clinicians, disagreed with the current optional nature of the policy, citing confusion over the best practices for their patients. However, they also did not express any preference toward a headgear mandate or a ban.

*“Some of the patients that I talk to, or even just other kids that I know playing lacrosse, it's the optional nature of it that has caused some distress for kids in terms of making that decision and what that decision might mean.” – Clinician*

*“That optional nature creates this kind of whirlwind of emotions, I think, for the kid, the family, whether it be positive, negative or neutral.” – Clinician*

Similarly, stakeholders also disagreed with the current optional nature of the policy. Conversely from the concussion experts however, many stakeholders felt headgear should be banned altogether, citing increased aggression towards and/or by players choosing to wear headgear as stated during the discussion related to arguments opposing headgear use.

## 4 Discussion

This qualitative study aimed to identify the need for and barriers to the development of mandated headgear use policies in girls’ lacrosse in Ohio. The present study gathered key information regarding the perceptions and thoughts on headgear use and mandates of both concussion experts and multiple stakeholder groups. Overall, both concussion experts and stakeholders reported an understanding of the potential consequences of concussion but did not perceive concussion as a pervasive problem in girls’ lacrosse. The most common argument in favor of headgear was the potential for increased player safety, specifically the prevention of head and facial injuries. However, the myriad of arguments discussed opposing mandated headgear use including increased aggressive play and/or targeting, concerns over changes in the game of girls’ lacrosse, and cost strongly outweighed the benefits in the eyes of the stakeholder. Finally, both concussion experts and stakeholders identified multiple organizations, such as USA Lacrosse and stakeholders (e.g., players, parents, and coaches) who could act as facilitators and/or barriers to the development, enactment, and implementation of policies related to headgear use in girls’ lacrosse.

Though members of the lacrosse community expressed an understanding of possible short and long-term consequences of concussion, most were not concerned about the incidence of concussion in girls’ lacrosse. This differs from recent findings from a 2023 study by Iyer and Bachynski (20) which found participants were concerned about concussion incidence, regardless of their stance on mandating headgear. Notably, the majority of the players included in the Iyer and Bachynski (20) study had experienced a concussion/TBI whereas only one of the nine players in the present study reported sustaining a concussion while playing lacrosse. Therefore, it is likely that prior experiences greatly contribute to concerns and awareness regarding concussion incidence and may not be generalizable across study samples.

Several arguments for opposing headgear use or a headgear mandate in girls’ lacrosse emerged from the stakeholder focus group discussions. Some of these arguments, such as headgear not preventing concussions and leading to increased aggression have been well covered in other work (7, 21). Concerns regarding increased aggression and targeting were commonly discussed among stakeholders in the present study. Towards these concerns, the design of girls’ lacrosse headgear leaves much of the head and face exposed, which should help mitigate the increased aggression known as the “gladiator effect” or Peltzman Effect (7, 22). This notion is somewhat supported by recent research which found that headgear did not reduce the number of impacts a player experienced but did reduce the magnitude of these impacts (23). However, given that stakeholders maintain strong beliefs that headgear will increase aggression and targeting, along with scepticism over the ability of headgear to prevent concussion in girls’ lacrosse, more research is needed to understand this possible relationship.

Similar to previous qualitative studies, another major argument for opposing mandated headgear use for girls was the differences between boys’ and girls’ lacrosse (20, 21). All stakeholder groups (i.e., players, parents, coaches, and officials) voiced concerns that the addition of headgear to the girls’ game would make it more similar to the boys’ game. Specifically, many of the players mentioned they did not mind the current level of aggression in a typical game, but they enjoyed that their game was different from the boys’ game and did not want it to become more aggressive or more similar to the boys’ game. While not all concussion experts were familiar with the specifics of girls’ lacrosse, those who were also expressed the importance of the differences between the two games. Furthermore, a more novel point related to the differences between the two sports was about the intricate hairstyles found in girls’ lacrosse. Players reported these hairstyles were important to build and maintain their team camaraderie and cited concerns that headgear would take away from this cultural aspect of the girls’ game. One way to address this concern may be developing transparent headgear to avoid obscuring these hairstyles.

Overall, focus group participants indicated headgear for girls’ lacrosse should be either mandated or banned, suggesting that the current optional policy may not be the optimal policy approach. This finding is in line with previous work which found that members of the lacrosse community were concerned that inconsistencies in rule implementation would pose a threat to player safety (20). Currently, USA Lacrosse states that the flexible headgear designed for girls’ lacrosse is allowable, but not mandatory for field players (4, 6). However, individual states are permitted to mandate the headgear if they choose, as was done in Florida in 2018. In Ohio, headgear continues to be optional, which was a topic of concern for both concussion experts and stakeholders. Though concussion experts did not explicitly express support for a mandate or a ban, they did not agree with the current optional nature of the headgear policy. Specifically, many clinicians stated the optional nature caused confusion and distress among their patients in deciding whether to wear headgear. Conversely, while stakeholders also disagreed with the current optional policy, many felt headgear should be banned for field players due to their beliefs of increased aggression and negative changes in girls’ game as a result of the policy. Although this viewpoint is different from those expressed in other qualitative work with college-level stakeholders in which stakeholders voiced support for the headgear to remain optional (21), both found that stakeholders would not support a headgear mandate.

Importantly, both concussion experts and stakeholders expressed the need for more data addressing the impact of headgear on head/facial injuries and musculoskeletal injuries and the levels of aggression and changes in officiating to be able to support a headgear mandate or similar policy development in girls’ lacrosse. Recent research examining injury data from high school girls’ lacrosse athletes via the NATION injury-surveillance methods, found that athletes who were required to wear headgear (e.g., high school teams in Florida) were at lower risk for concussion than those who were not required to wear headgear (e.g., high school teams in other states) and did not have any increased risk of musculoskeletal injury (12, 13). Though the study suggests headgear has the potential to reduce concussion risk among high school girls’ lacrosse athletes, very few stakeholders were aware of this evidence while others expressed their need for additional information or had questions about the study and its implications. Considering this, dissemination of existing and future research evidence on this topic to stakeholders is imperative to lay the groundwork for policy development related to headgear in girls’ lacrosse.

There were some limitations to the present study. First, the study included only concussion experts and girls’ lacrosse stakeholders in the state of Ohio, primarily involved in high school girls’ lacrosse, therefore the data gathered may not reflect the views of those from other states or other levels of girls’/women's lacrosse (e.g., middle school, college). Further research using both qualitative and quantitative approaches across multiple states and levels of play is needed to obtain a more complete picture of where the girls’/women's lacrosse community stands on this controversial issue. Specifically, quantitative-focused studies, such as randomized controlled trials, similar to past work done in rugby and soccer (24, 25), to assess the effectiveness of headgear in reduction of head injuries are needed. Additionally, recent evaluations of body-checking policies in youth ice hockey in Canada, showing lower injury rates in non-body checking leagues, may serve as a model for headgear policy development and implementation in girls’ lacrosse (26–29). Moreover, additional mixed methods studies to better understand the views of various groups of lacrosse community members are needed. Finally, conformity bias may have influenced the thoughts shared and direction of the focus group discussions. However, players were separated from their parents and coaches and the focus group facilitators used various techniques, such as probing for clarification and directing questions towards participants who had not yet shared, to ensure the opinions of all participants were heard and thoughtfully explored.

## 5 Conclusion

Overall, concussion experts and stakeholders identified possible reasons for headgear use related to injury prevention but also identified several important barriers to the development of a headgear mandate for girls’ lacrosse. Reasonings for their opposition to headgear use or a mandate centered around safety concerns due to increased aggression, changes to aspects of the game that make girls’ lacrosse a unique sport, changes to the culture of the sport, and concerns regarding increased cost. Well-disseminated research addressing the impact of headgear on head/facial injuries and concussions, levels of aggression, and officiating practices are needed to gain support for a headgear mandate.

## Data Availability

The raw data supporting the conclusions of this article will be made available by the authors, without undue reservation.
